# Supplementary figures and images for: Vertical assemblage of the holoplanktonic mollusks (Pteropoda and Pterotracheoidea: Carinaiidae, Pterotracheidae) in the Campeche Canyon, southern Gulf of Mexico, during a “Nortes” season
Source: PeerJ. 2025 Mar 31;13:e19118. doi: 10.7717/peerj.19118 (PMC11967430; doi:10.7717/peerj.19118)

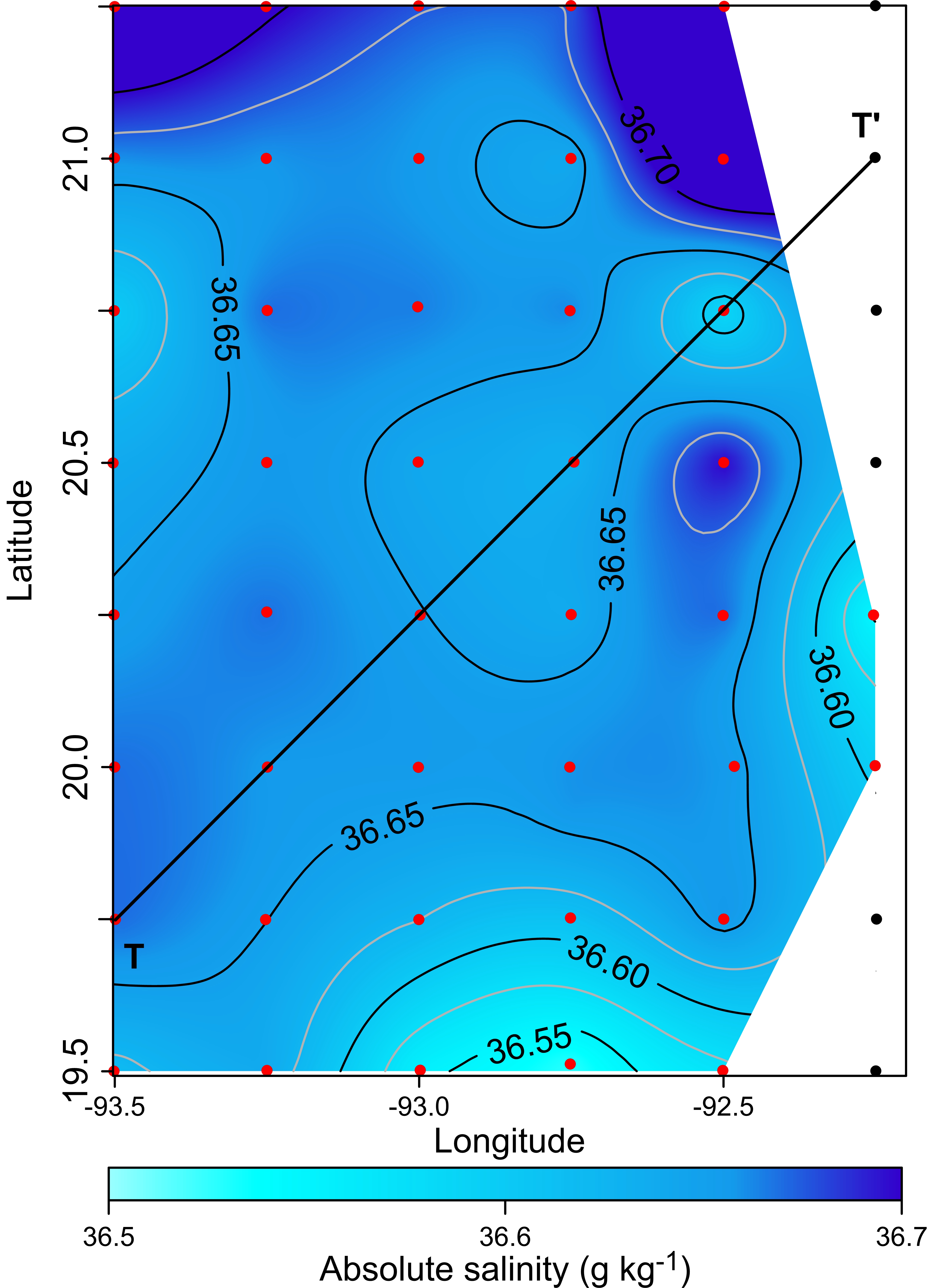

Supplement: Supplemental Information 1 [file peerj-13-19118-s001.jpg]

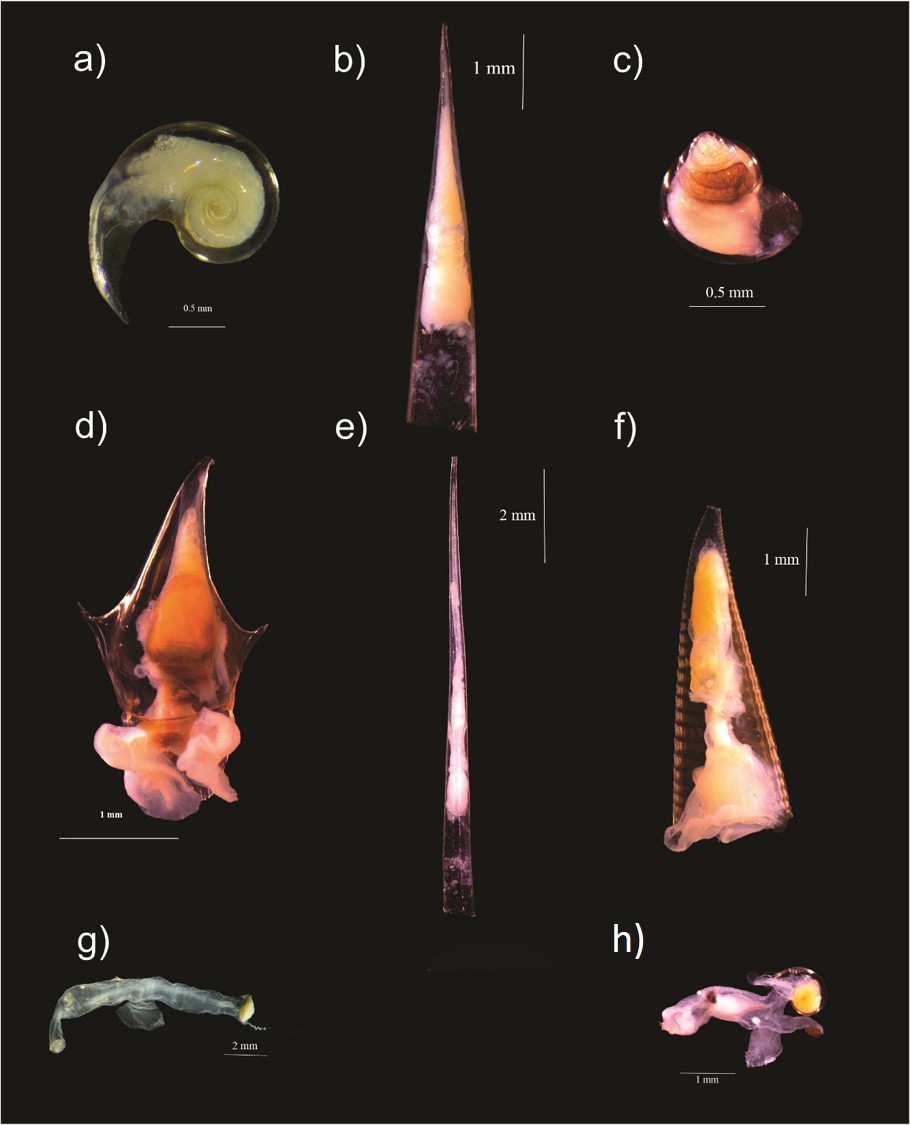

Supplement: Supplemental Information 2 — a) Heliconoides inflatus (A. d’Orbigny, 1835), b) Creseis conica Eschscholtz, 1829, c) Limacina trochiformis (A. d’Orbigny, 1835), d) Cavolinia inflexa (Lesueur, 1813), e) Creseis acicula (Rang, 1828); y Superfamily Pterotracheoidea (Carinariidade, Pterotrachidae): f) Hyalocylis striata (Rang, 1828), g) Firoloida desmarestia Lesueur, 1817, h) Carinaria pseudorugosa Vayssière, 1904, In the Campeche Canyon during ”Nortes” season. [file peerj-13-19118-s002.jpg]
