# Supplementary material for: Vertical assemblage of the holoplanktonic mollusks (Pteropoda and Pterotracheoidea: Carinaiidae, Pterotracheidae) in the Campeche Canyon, southern Gulf of Mexico, during a “Nortes” season
Source: PeerJ. 2025 Mar 31;13:e19118. doi: 10.7717/peerj.19118 (PMC11967430; doi:10.7717/peerj.19118)
Supplement: Supplemental Information 5 [file peerj-13-19118-s005.docx]

| Station | Total depth (m) | Date | Hour (Local time) | Latitude | Longitude |
| --- | --- | --- | --- | --- | --- |
| 1 | 2837.2 | 22-02-11 | 21:33 | 21.25 | -93.50 |
| 2 | 2453.19 | 23-02-11 | 1:30 | 21.25 | -93.25 |
| 3 | 2906.65 | 23-02-11 | 3:53 | 21.25 | -93.00 |
| 4 | 2878.74 | 23-02-11 | 7:10 | 21.25 | -92.75 |
| 5 | 661.4 | 23-02-11 | 9:20 | 21.25 | -92.50 |
| 6 | 65.3 | 23-02-11 | 12:27 | 21.25 | -92.25 |
| 7 | 53.70 | 23-02-11 | 14:32 | 21.00 | -92.25 |
| 8 | 1372.96 | 23-02-11 | 16:26 | 21.00 | -92.50 |
| 9 | 2422.54 | 23-02-11 | 18:30 | 21.00 | -92.75 |
| 10 | 2546.98 | 23-02-11 | 21:14 | 21.00 | -93.00 |
| 11 | 2655 | 23-02-11 | 23:28 | 21.00 | -93.25 |
| 12 | 2076.76 | 24-02-11 | 2:10 | 21.00 | -93.50 |
| 13 | 2315.85 | 24-02-11 | 4:25 | 20.75 | -93.50 |
| 14 | 1886.31 | 24-02-11 | 7:26 | 20.75 | -93.25 |
| 15 | 2239.11 | 24-02-11 | 10:16 | 20.76 | -93.00 |
| 16 | 2645.49 | 24-02-11 | 14:15 | 20.75 | -92.75 |
| 17 | 1909.32 | 24-02-11 | 16:52 | 20.75 | -92.50 |
| 18 | 52.71 | 24-02-11 | 19:37 | 20.75 | -92.25 |
| 19 | 56 | 24-02-11 | 21:40 | 20.50 | -92.25 |
| 20 | 2370 | 24-02-11 | 23:46 | 20.50 | -92.50 |
| 21 | 1757 | 25-02-11 | 2:04 | 20.50 | -92.75 |
| 22 | 1542 | 25-02-11 | 4:45 | 20.50 | -93.00 |
| 23 | 1732.77 | 25-02-11 | 6:52 | 20.50 | -93.25 |
| 24 | 1785.31 | 25-02-11 | 9:32 | 20.50 | -93.50 |
| 25 | 1725.63 | 25-02-11 | 12:05 | 20.25 | -93.50 |
| 26 | 1251.99 | 25-02-11 | 15:01 | 20.26 | -93.25 |
| 27 | 1123.73 | 25-02-11 | 17:19 | 20.25 | -93.00 |
| 28 | 1621.7 | 25-02-11 | 20:12 | 20.25 | -92.75 |
| 29 | 1940 | 25-02-11 | 22:34 | 20.25 | -92.50 |
| 30 | 505.99 | 26-02-11 | 1:32 | 20.25 | -92.25 |
| 31 | 77.82 | 26-02-11 | 4:10 | 20.00 | -92.25 |
| 32 | 1332.97 | 26-02-11 | 6:33 | 20.00 | -92.48 |
| 33 | 1118.5 | 26-02-11 | 8:40 | 19.93 | -92.75 |
| 34 | 1306 | 26-02-11 | 11:29 | 20.00 | -93.00 |
| 35 | 1287.33 | 26-02-11 | 13:30 | 20.00 | -93.25 |
| 36 | 826.97 | 26-02-11 | 16:15 | 20.00 | -93.50 |
| 37 | 1195.9 | 26-02-11 | 18:12 | 19.75 | -93.50 |
| 38 | 1091 | 26-02-11 | 21:01 | 19.75 | -93.25 |
| 39 | 886 | 26-02-11 | 23:18 | 19.75 | -93.00 |
| 40 | 949.93 | 27-02-11 | 2:09 | 19.75 | -92.75 |
| 41 | 275.59 | 27-02-11 | 4:20 | 19.75 | -92.50 |
| 42 | 107.61 | 27-02-11 | 6:45 | 19.75 | -92.25 |
| 43 | 65.19 | 27-02-11 | 8:15 | 19.50 | -92.25 |
| 44 | 132.89 | 27-02-11 | 10:28 | 19.50 | -92.50 |
| 45 | 225.55 | 27-02-11 | 12:00 | 19.51 | -92.75 |
| 46 | 859.5 | 27-02-11 | 13:35 | 19.50 | -93.00 |
| 47 | 583 | 27-02-11 | 15:38 | 19.50 | -93.25 |
| 48 | 673.08 | 27-02-11 | 14:21 | 19.50 | -93.50 |
